# Longitudinal blood pressure trajectories and tau‐lipid biomarkers associated with dementia in hypertensive adults: Results from the population‐based CRHCP cohort

**DOI:** 10.1002/alz.71346

**Published:** 2026-04-06

**Authors:** Shanshan Zhong, Xiaofan Guo, Nanxiang Ouyang, Lanqing Zhao, Jinwei Li, Fangxi Liu, Chang Wang, Weishuang Xue, Zhijia Liang, Yingxian Sun, Mei Zhao, Chuansheng Zhao

**Affiliations:** ^1^ Department of Neurology The First Hospital of China Medical University Shenyang Liaoning China; ^2^ Key Laboratory of Neurological Disease Big Data of Liaoning Province Shenyang Liaoning China; ^3^ Shenyang Clinical Medical Research Center for Difficult and Serious Diseases of the Nervous System Shenyang Liaoning China; ^4^ Department of Cardiology The First Hospital of China Medical University Shenyang Liaoning China; ^5^ Department of Sleep Medicine Center Shengjing Hospital of China Medical University Shenyang Liaoning China; ^6^ Department of Cardiology Shengjing Hospital of China Medical University Shenyang Liaoning China

**Keywords:** blood pressure trajectory, composite indices, dementia, hypertension, longitudinal cohort, metabolic dysregulation, pTau217, public health

## Abstract

**INTRODUCTION:**

Blood pressure (BP) trajectories are associated with dementia, but their relevance across neurodegenerative and metabolic backgrounds in hypertensive populations remains unclear.

**METHODS:**

We analyzed 28,135 hypertensive participants from the China Rural Hypertension Control Project to estimate BP trajectories over 4 years using latent class group‐based models. A nested case–control study was designed to assess interaction effects between BP trajectories and pTau217‐lipid composite indices on dementia.

**RESULTS:**

Four BP trajectories were identified. In fully adjusted models, the high‐stable diastolic BP trajectory showed the strongest association with dementia (odds ratio [OR] = 2.14, 95% confidence interval [CI] 1.74–2.63), followed by high‐stable systolic BP trajectory (OR = 1.75, 95% CI 1.42–2.15). In the nested case‐control analysis, significant interactions between pTau217‐lipid composite indices and BP trajectories (high‐declining and moderate‐stable) were observed in relation to dementia.

**DISCUSSION:**

High‐stable BP trajectories were consistently associated with dementia, whereas the associations between high‐declining and moderate‐stable BP trajectories varied according to pTau217‐lipid composite indices.

## Highlights

- High‐stable systolic and diastolic blood pressure (BP) trajectories are consistently associated with dementia in older hypertensive adults.
- The associations of high‐declining and moderate‐stable BP trajectories with dementia are modified by serum pTau217‐lipid composite indices.
- Integration of serum pTau217‐lipid composite indices with longitudinal BP trajectories enhances dementia stratification.

## Graphical abstract

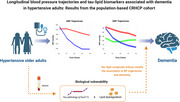

## 1 BACKGROUND

Dementia is a growing public health concern and has become one of the leading causes of mortality worldwide.^1^ Hypertension is a major modifiable risk factor^2^, ^3^, ^4^; however, the relationship between blood pressure (BP) and dementia remains complex, particularly in older adults.^5^, ^6^ Moreover, reliance on BP measurements obtained at a single time point may inadequately capture the dynamic patterns of BP.

Accordingly, longitudinal cohort studies have examined BP trajectories across midlife and late life in relation to dementia, and the evidence to date remains inconsistent.^7^, ^8^ Midlife BP patterns have been linked to dementia in later life. In the Framingham Heart Study, cumulative BP exposure across midlife was reported to be associated with dementia after age 65.^9^ In cohorts of 1890 men followed for up to 32 years and 707 women followed for up to 37 years, declining systolic BP (SBP) trajectories from midlife to late life were shown to precede the clinical onset of dementia.^10^, ^11^ In late life, several cohorts including the Kungsholmen project have reported that changes in BP trajectories, particularly declining SBP and/or diastolic BP (DBP) were associated with dementia.^7^, ^12^, ^13^ Additional studies have indicated that longitudinal increase in SBP was associated with dementia.^14^, ^15^ However, the Chicago Health and Aging Project did not observe a significant association between elevated BP and Alzheimer's disease.^16^ These discrepancies may reflect heterogeneity in study design, including sample size, BP measurement frequency, and analytic approaches. In prior studies, BP was often measured at prolonged intervals, limiting the capture of fine‐grained trajectory patterns and short‐term changes.

Moreover, most existing studies were conducted in general population‐based cohorts. Although some investigations performed subgroup analyses among individuals with baseline hypertension, these analyses suggested that the associations between BP trajectories and dementia in hypertensive populations differed substantially from those observed in the overall population.^11^, ^13^ However, evidence specifically characterizing BP trajectories in hypertensive populations remains limited. To date, no large‐scale longitudinal studies have examined the association between BP trajectories and dementia specifically in hypertensive populations. Addressing this gap could enhance our understanding of BP trajectory patterns and improve dementia stratification in hypertensive individuals.

Beyond vascular factors, metabolic dysfunctions are increasingly recognized as important contributors to dementia.^17^, ^18^ Accumulating evidence has demonstrated that impaired lipid homeostasis is closely involved in the onset and progression of dementia.^19^ Notably, lipid dysregulation may modulate tau‐related neurofibrillary pathology and downstream neurodegenerative processes.^20^ While prior studies have explored interactions between BP trajectories and demographic factors such as age and sex, the extent to which baseline metabolic dysfunction and neurodegenerative burden modify the association between BP trajectories and subsequent dementia remains unclear. Phosphorylated tau at threonine 217 (pTau217), a blood‐based biomarker reflecting tau pathology, has emerged as one of the most specific and robust markers of Alzheimer's‐related neurodegeneration.^21^, ^22^ The availability of blood‐based pTau217 measurements has enabled assessment of neurodegenerative burden in large cohort studies, including retrospective analyses using archived baseline samples.

In the present study, we leveraged the longitudinal data from the China Rural Hypertension Control Project (CRHCP), a cluster‐randomized trial.^23^ Focusing on long‐term BP trajectories, metabolic dysfunction, neurodegenerative burden, and dementia, our study aimed to: (1) explore the associations between BP trajectories and dementia in a hypertensive population; (2) assess whether the impact of BP trajectories on dementia is influenced by baseline metabolic and neurodegenerative burden; and (3) evaluate the accuracy of integrating BP trajectories and pTau217‐lipid composite indices for dementia discrimination. We hypothesize that (1) BP trajectories are associated with dementia in hypertensive individuals, and (2) this association is shaped by underlying neurodegenerative and metabolic vulnerability.

**RESEARCH IN CONTEXT**

1. **Systematic review**: Previous studies have linked blood pressure (BP) trajectories with dementia, but findings have been inconsistent, and large‐scale longitudinal evidence in hypertensive adults remains limited. In particular, the modifying roles of neurodegenerative and metabolic factors in this association have not been systematically examined.
2. **Interpretation**: High‐stable systolic and diastolic BP trajectories were consistently associated with dementia, whereas the associations of high‐declining and moderate‐stable BP trajectories varied according to pTau217‐lipid composite indices.
3. **Future directions**: Our findings provide a framework for future studies on dementia management in older hypertensive adults, highlighting the potential of integrating BP trajectory monitoring with neurodegenerative and lipid metabolic biomarkers to improve risk stratification and guide personalized prevention strategies.

## 2 MATERIALS AND METHODS

### 2.1 Study design

**FIGURE 1:**
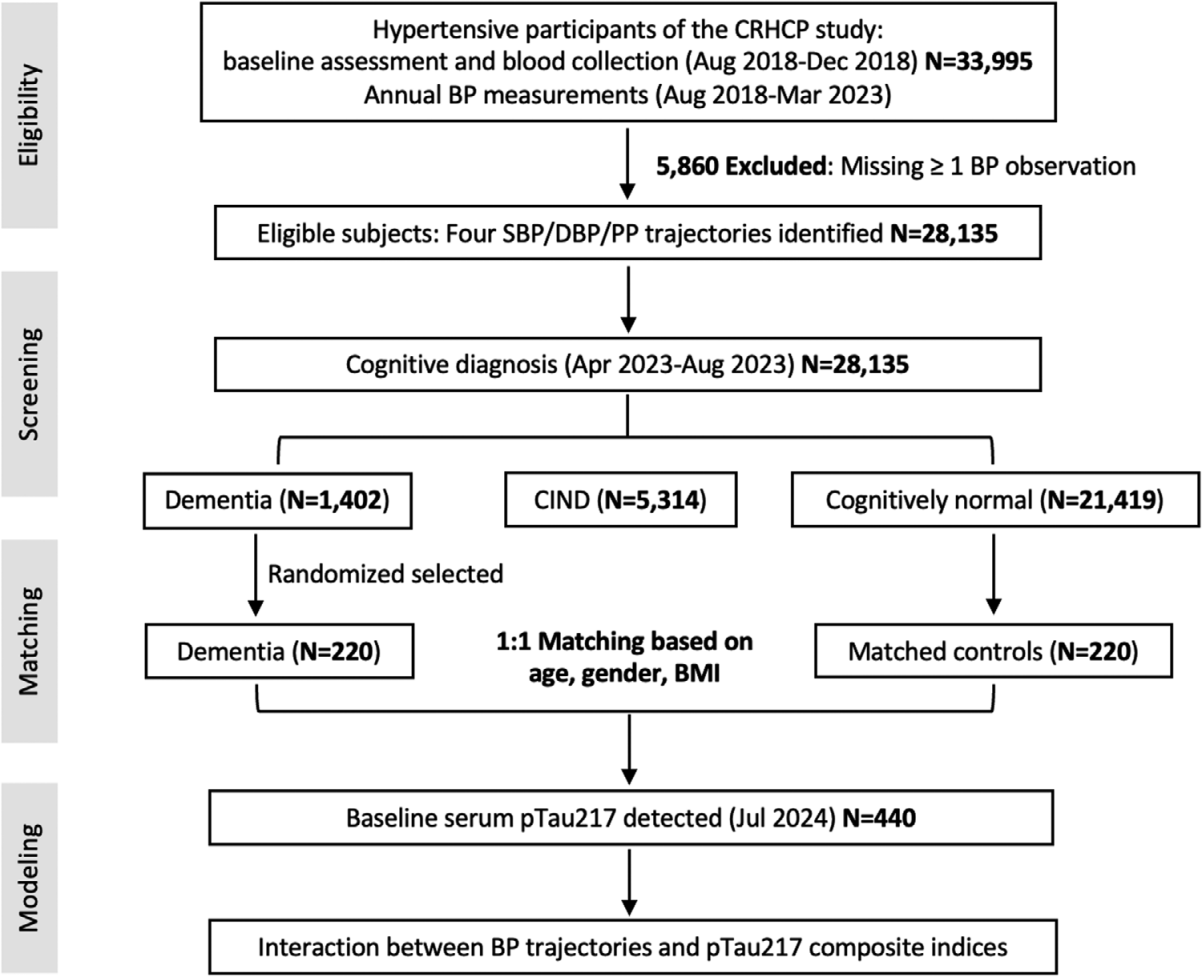
Study flowchart and analytical framework. Participant selection and exclusions, blood pressure trajectory modeling, cognitive assessment, and nested case‐control sampling for biomarker analyses are presented. BMI, body mass index; BP, blood pressure; CIND, cognitive impairment no dementia; CRHCP, China Rural Hypertension Control Project; DBP, diastolic blood pressure; PP, pulse pressure; SBP, systolic blood pressure.

CRHCP, an open‐label, blinded‐endpoint, cluster‐randomized trial was conducted between August 2018 and March 2023, involving 33,995 adults with hypertension from 326 rural villages in China.^23^ Among these, 28,135 individuals with complete BP measurements across five visits during the 4‐year follow‐up period were included in the final analysis; 5860 were excluded due to missing ≥ 1 BP observation. Analyses were performed in two sequential steps: First, longitudinal BP trajectories were characterized in the analytical cohort of 28,135 participants using data from all five follow‐up visits. Dementia status was systematically evaluated at the end of the follow‐up period using standardized clinical assessments and neurologist adjudication. Second, a nested case–control study was conducted within this cohort to examine whether baseline tau pathology modifies the association between BP trajectories and dementia. Among the total of 1402 dementia diagnosed at the end of the follow‐up, a retrospective screening of their baseline cognitive function and stroke history was performed. Finally, 220 dementia cases were randomly selected and matched in a 1:1 ratio with 220 cognitively normal controls with adjustment on age, sex, and body mass index. All participants included in the nested case‐control analysis had normal cognitive function at baseline and had no history of stroke at baseline or incident stroke during the follow‐up. BP trajectory group assignments identified in the full cohort were applied to participants in the nested case‐control study. In addition, baseline serum pTau217 and metabolic biomarkers were measured to enable dementia stratification and effect modification analyses. The overall study workflow is illustrated in **Figure** **1**. All participants provided written informed consent prior to inclusion in the study. The study was conducted in accordance with the ethical principles outlined in the Declaration of Helsinki and local regulatory guidelines. Additionally, this study was approved by the Institutional Ethics Committee of the First Hospital of China Medical University and registered at ClinicalTrials.gov (Identifier: NCT03527719).

### 2.2 Data collection and variables

Baseline characteristics were collected using a combination of self‐reported questionnaires and laboratory measurements. Self‐reported variables included education, anti‐hypertensive medications, lifestyle factors such as smoking status, alcohol consumption, and physical activities. Additionally, participants reported their medical history, including diagnosis of diabetes, chronic kidney disease, and cardiovascular disease. These self‐reported data were validated through follow‐up interviews and clinical records where possible. Laboratory data were obtained through standardized tests conducted by trained personnel. BP measurements were taken at each visit using a standardized protocol with calibrated devices. Serum biomarkers, including total cholesterol (TC), triglycerides (TG), low‐density lipoprotein cholesterol (LDL‐C), high‐density lipoprotein cholesterol (HDL‐C), creatinine, uric acid, blood urea nitrogen, alanine aminotransferase, aspartate aminotransferase, and glucose were measured at baseline using high‐sensitivity assays. These laboratory tests were conducted in accredited clinical laboratories, with quality control measures in place to ensure the accuracy and reliability of the results.

### 2.3 BP trajectories identification

#### 2.3.1 Definition of BP trajectories

The CRHCP cohort was initiated with standardized baseline assessments, followed by repeated BP measurements collected at predefined time points over a 4‐year period using a standardized protocol by trained staff.^23^ Longitudinal trajectories of SBP, DBP, and pulse pressure (PP) were defined using repeated annual BP measurements at baseline (year 0) and during 4 years of follow‐up (years 1–4).

#### 2.3.2 Modeling of BP trajectories

BP trajectories were modeled using latent class group‐based modeling (LCGBM), a semi‐parametric approach that classifies individuals into distinct latent groups according to similar longitudinal BP patterns over time. Separate models were fitted for SBP, DBP, and PP. For each BP component, unconditional models (without covariates) were estimated with two to six latent classes. Linear, quadratic, and cubic polynomial terms were evaluated to allow for different shapes of the trajectories. Model selection was guided by multiple criteria, including lower values of the log‐likelihood, Akaike Information Criterion (AIC), Bayesian Information Criterion (BIC), and sample‐size–adjusted BIC (SSA‐BIC), as well as higher entropy values indicating better classification accuracy. The Lo–Mendell–Rubin adjusted likelihood ratio test and bootstrap likelihood ratio test were used to compare models with k versus (k‐1) classes.

#### 2.3.3 Categorization of BP trajectories

The optimal number of BP trajectory groups was determined based on overall model fit, classification quality, and clinical interpretability, with the additional constraint that the smallest trajectory group contained at least 1.0% of the study population. Individuals were assigned to trajectory groups according to the maximum posterior probability derived from the fitted LCGBM. Trajectory groups were subsequently labeled descriptively based on their longitudinal BP patterns over the follow‐up period.

### 2.4 Outcome measurements

After completion of the BP trajectory assessment, dementia outcomes were systematically evaluated using standardized diagnostic criteria determined by neurologist adjudication based on the National Institute on Aging‐Alzheimer's Association criteria.^24^, ^25^, ^26^ The study records of medical history, neurological examination findings, and cognitive assessments (Mini‐Mental State Examination,^27^, ^28^ Functional Activities Questionnaire,^29^, ^30^, ^31^ and Quick Dementia Rating System^32^) were used to adjudicate cognitive status. These three scales have been translated into Chinese and validated in the Chinese population.^33^, ^34^, ^35^ All neurologists responsible for cognitive assessments received standardized training in neuropsychological evaluation from a board‐certified clinical neuropsychologist with extensive experience in dementia diagnosis. Each case was independently reviewed by two adjudicators using standardized diagnostic criteria for all‐cause dementia. If the two reviewers agree on the diagnosis, it was recorded as the final diagnosis. If the two reviewers disagree, a third and more experienced adjudicator participated in review and discussion to reach consensus. Dementia subtypes were not classified.

### 2.5 Serum pTau217 measurements

Fasting blood samples were collected at baseline in 2018 according to standardized protocols, processed to obtain serum, and stored at ‐80°C until analysis. Serum pTau217 concentrations were measured in July 2024, using the archived baseline serum samples. Measurement was conducted using a validated single‐molecule array immunoassay (Iomics Biosciences), employing monoclonal antibodies specific for pTau217. All serum samples were analyzed in duplicate, with operators blinded to clinical outcomes to minimize measurement bias. Calibration of the assay was conducted using recombinant pTau217 standards, ensuring accurate quantification of pTau217 concentrations. The inter‐assay and intra‐assay coefficients of variation were both < 10%, ensuring high reproducibility and reliability of the assay.

#### 2.5.1 pTau217 composite indices measurements

A series of pTau217‐based composite indices were constructed to characterize the combined influence of neurodegenerative burden and lipid dysfunction. These indices integrated baseline serum pTau217 with established lipid metabolism markers, which have been widely reported and individually associated with dementia.^36^, ^37^, ^38^, ^39^ All parameters were measured from the same baseline serum sample. To examine potential effect modification by lipid metabolism, multiplicative interaction terms between pTau217, lipid indices and BP trajectories were specified a priori in the regression models. The following composite indices were derived:

1. pTau217 and TC to HDL‐C Ratio Interaction^36^ (pTau217−TC/HDL−C) = pTau217 × (TC/HDL−C).
2. pTau217 and LDL‐C to HDL‐C Ratio Interaction^37^ (pTau217−LDL−C/HDL−C) = pTau217 × (LDL−C/HDL−C).
3. pTau217 and NonHDL‐C Interaction^38^ = pTau217 × (TC−HDL−C).

The following units were used for the composite indices: pTau217 was measured in pg/mL; TC, LDL‐C, and HDL‐C in mmol/L.

### 2.6 Statistical analyses

In the full cohort, generalized linear mixed models (GLMMs) were used to assess the association between BP trajectories and dementia. The GLMMs had a binary distribution for the occurrence of dementia and logit link function, BP trajectory class as the study variable with the lowest trajectory (class 4) as the reference and village as random effect, adjusting for CRHCP randomization arm, demographic characteristics, lifestyle and socioeconomic factors, antihypertensive medication use, major vascular comorbidities, and baseline metabolic and laboratory parameters, from which odds ratios and 95% confidence intervals (CIs) were derived. For the nested case‐control data analysis to assess the effect of BP trajectory class on dementia outcomes, an additional random effect for pair was added into the above GLMMs. Effect modifications of BP trajectories by serum pTau217 and metabolic factors were evaluated by including interaction terms between BP trajectories with dichotomized serum pTau217 and metabolic factors in the above GLMMs. In the interaction analyses, continuous exposure variables were dichotomized using their median values to ensure stable estimates of BP trajectory effects. To evaluate the discriminative performance of baseline biomarkers and composite indices for dementia stratification, receiver operating characteristic (ROC) curve analyses were performed.^40^ Sequential models were evaluated, including a base model with demographic variables, models incorporating BP trajectories, and models further augmented with serum pTau217 and pTau217‐based composite indices. The predicted probabilities derived from each model were used to construct ROC curves and calculate the area under the ROC curve (AUROC). Differences in AUROC values between models were compared using DeLong's test. Sensitivity analyses were conducted by re‐estimating BP trajectories within the nested case‐control sample using the same LCGBM approach to assess the robustness of the main findings. Continuous variables are presented as mean (standard deviation) or median (interquartile range), as appropriate, and were compared using *t*‐tests or analysis of variance. Categorical variables are presented as counts (percentages) and were compared using chi‐square tests. Statistical analyses were performed using R software (version 4.3.1).

## 3 RESULTS

### 3.1 Baseline characteristics and BP trajectories identification

**FIGURE 2:**
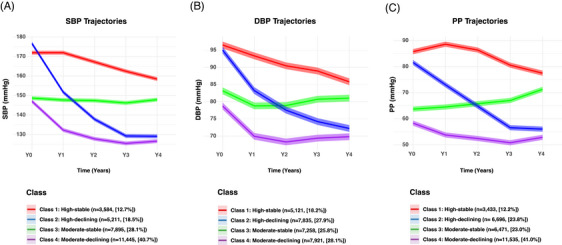
Latent class growth‐based trajectories of BP over 4 years in the full cohort. Latent class growth‐based modeling identified four distinct trajectories for SBP, DBP, and PP over 4 years of follow‐up. (A) SBP trajectories. (B) DBP trajectories. (C) PP trajectories. Solid lines represent class‐specific model‐predicted trajectories, and shaded areas indicate 95% confidence intervals. BP was measured at baseline (Y0) and annually thereafter (Y1–Y4). Four trajectory patterns were identified for each BP component: (1) high‐stable; (2) high‐declining; (3) moderate‐stable, and (4) moderate‐declining. BP, blood pressure; DBP, diastolic blood pressure; PP, pulse pressure; SBP, systolic blood pressure.

In the full analytical CRHCP cohort (*N* = 28,135), the mean (SD) age was 67.0 (8.9) years, and 61.8% were women (**Table** **1**). Using repeated BP measurements, model fit statistics for two‐ to six‐class LCGBM for SBP, DBP, and PP are presented in **Tables** S1‐S3. Based on these criteria, a four‐class solution was selected for each BP component: class 1 (high‐stable), class 2 (high‐declining), class 3 (moderate‐stable), and class 4 (moderate‐declining) (**Figure** **2**). Baseline characteristics by trajectory class are shown in Tables S4 and S5.

**TABLE 1:** Baseline characteristics of participants in the full cohort.

Characteristics	Total (*n* = 28,135)
Mean age, years	67.0 (8.9)
Female sex, *n* (%)	17,401 (61.8%)
Mean BMI, kg/m^2^ (SD)	26.04 (3.8)
Less than primary school, *n* (%)	5695 (20.2%)
Current smoking, *n* (%)	5157 (18.3%)
History of stroke, *n* (%)	4903 (17.4%)
History of diabetes, *n* (%)	2406 (8.5%)
History of CKD, *n* (%)	159 (0.5%)
Mean TC, mmol/L (SD)	5.04 (1.0)
Mean TG, mmol/L (SD)	1.98 (1.6)
Mean LDL‐C, mmol/L (SD)	2.71 (0.8)
Mean HDL‐C, mmol/L (SD)	1.44 (0.3)
Mean plasma glucose, mmol/L (SD)	6.15 (2.0)
Mean ALT, U/L (SD)	22.07 (15.8)
Mean AST, U/L (SD)	22.12 (11.2)
Mean total protein, g/L (SD)	73.85 (5.1)
Mean uric acid, µmol/L (SD)	301.7 (85.3)
Mean serum creatinine, mg/dL (SD)	0.90 (0.2)
Mean BUN, mmol/L (SD)	5.47 (2.0)
Mean antihypertensive medications (SD)	1.96 (1.5)
Mean SBP, mmHg (SD)	156.0 (17.5)
Mean DBP, mmHg (SD)	87.7 (10.5)
Mean PP, mmHg (SD)	68.3 (15.8)

*Note*: This table presents baseline characteristics of participants in the full cohort. Values are reported as mean (SD) for continuous variables and *n* (%) for categorical variables.

Abbreviations: ALT, alanine aminotransferase; AST, aspartate aminotransferase; BMI body mass index; BUN, blood urea nitrogen; CKD, chronic kidney disease; DBP, diastolic blood pressure; HDL‐C, high‐density lipoprotein cholesterol; LDL‐C, low‐density lipoprotein cholesterol; PP, pulse pressure; SD, standard deviation; SBP, systolic blood pressure; SD, standard deviation; TC, total cholesterol; TG, triglyceride.

### 3.2 Associations of BP trajectories with dementia

In the full cohort, associations between BP trajectories and dementia were assessed with class 4 (moderate‐declining) as the reference in sequentially adjusted models. In the fully adjusted model, DBP class 1 showed the strongest association with dementia (OR = 2.14, 95% CI 1.74–2.63), followed by SBP class 1 (OR = 1.75, 95% CI 1.42–2.15), SBP class 2 (OR = 1.44, 95% CI 1.21–1.71), DBP class 3 (OR = 1.31, 95% CI 1.10–1.56), and DBP class 2 (OR = 1.25, 95% CI 1.05–1.49). However, PP trajectories were not significantly associated with dementia after adjustments (**Table** **2**).

**TABLE 2:** Association between BP trajectories and dementia from generalized linear mixed models.

Parameter	SBP class 1	SBP class 2	SBP class 3	SBP class 4
Total population	3584 (12.74%)	5211 (18.52%)	7895 (28.06%)	11,445 (40.68%)
Dementia, *n* (%)	277 (7.73%)	321 (6.16%)	381 (4.83%)	423 (3.70%)
Model 1	2.66 (2.24–3.16)	1.87 (1.60–2.18)	1.46 (1.25–1.70)	1
Model 2	1.81 (1.50–2.19)	1.42 (1.21–1.68)	1.26 (1.06–1.49)	1
Model 3	1.76 (1.45–2.15)	1.41 (1.20–1.67)	1.23 (1.03–1.46)	1
Model 4	1.76 (1.43–2.16)	1.44 (1.21–1.71)	1.19 (0.99–1.43)	1
Model 5	**1.75 (1.42**–**2.15)**	**1.44 (1.21**–**1.71)**	1.18 (0.98–1.41)	1

	**DBP class 1**	**DBP class 2**	**DBP class 3**	**DBP class 4**
Total population	5121 (18.20%)	7835 (27.85%)	7258 (25.80%)	7921 (28.15%)
Dementia, *n* (%)	262 (5.12%)	300 (3.83%)	393 (5.41%)	447 (5.64%)
Model 1	0.82 (0.69–0.96)	0.61 (0.52–0.71)	0.91 (0.78–1.05)	1
Model 2	2.14 (1.77–2.59)	1.26 (1.07–1.50)	1.33 (1.13–1.56)	1
Model 3	2.11 (1.73–2.56)	1.24 (1.05–1.47)	1.31 (1.11–1.55)	1
Model 4	2.10 (1.71–2.58)	1.23 (1.03–1.47)	1.30 (1.09–1.54)	1
Model 5	**2.14 (1.74**–**2.63)**	**1.25 (1.05**–**1.49)**	**1.31 (1.10**–**1.56)**	1

	**PP class 1**	**PP class 2**	**PP class 3**	**PP class 4**
Total population	3424 (12.17%)	6687 (23.77%)	6468 (22.99%)	11,556 (41.07%)
Dementia, *n* (%)	272 (7.94%)	427 (6.39%)	309 (4.78%)	394 (3.41%)
Model 1	3.16 (2.67–3.75)	2.19 (1.89–2.53)	1.58 (1.34–1.85)	1
Model 2	1.11 (0.91–1.34)	1.02 (0.87–1.19)	1.01 (0.85–1.20)	1
Model 3	1.11 (0.91–1.35)	1.03 (0.88–1.22)	1.00 (0.84–1.19)	1
Model 4	1.09 (0.89–1.34)	1.01 (0.85–1.19)	0.94 (0.78–1.13)	1
Model 5	1.09 (0.89–1.34)	1.00 (0.85–1.18)	0.93 (0.77–1.12)	1

*Note*: Models estimated the associations between BP trajectory classes and dementia, with class 4 as the reference group. ORs and 95% CIs were derived from generalized linear mixed models with a random intercept for village to account for clustering at the village level. Model 1 included BP trajectory classes only. Model 2 was additionally adjusted for intervention assignment, age, sex, body mass index, and education. Model 3 further adjusted for income, physical activity, antihypertensive medication use, alcohol consumption, and cigarette smoking. Model 4 additionally adjusted for history of stroke, myocardial infarction, diabetes, and chronic kidney disease. Model 5 further adjusted for baseline total cholesterol, triglycerides, LDL‐C, HDL‐C, glucose, alanine aminotransferase, aspartate aminotransferase, total protein, uric acid, creatinine, and blood urea nitrogen.

Abbreviations: BP, blood pressure; DBP, diastolic blood pressure CI, confidence interval; OR, odds ratio; PP, pulse pressure; SBP, systolic blood pressure

### 3.3 Characterization of the nested case‐control study population

To investigate whether the degree of neurodegenerative pathology at baseline influences the association between BP trajectories and dementia, we conducted a nested case‐control study within the CRHCP trial, including 220 dementia cases and 220 matched controls. The baseline mean (SD) age was 75.5 (7.4) years, all of whom were cognitively normal at baseline and stroke‐free during the 4 years of follow‐up. Sensitivity analyses demonstrated that re‐estimation of BP trajectories within the nested case‐control sample reproduced four BP trajectory patterns comparable to those observed in the full cohort (**Figure** **S1**). Baseline characteristics of these trajectory classes were shown in **Tables** S6 and S7. Individuals with dementia had significantly higher baseline serum pTau217 concentrations (0.224 pg/mL vs. 0.161 pg/mL; *P* = 0.012) (**Table** **3**).

**TABLE 3:** Baseline characteristics of dementia and cognitively normal controls in the nested case‐control study.

Parameter	Cognitively normal (*n* = 220)	Dementia (*n* = 220)	*p*‐value
Mean age, years (SD)	75.30 (6.7)	76.14 (7.3)	0.331
Mean BMI, kg/m^2^ (SD)	24.91 (3.7)	24.62 (3.8)	0.363
Female sex, *n* (%)	178 (80.9%)	178 (80.9%)	1.000
Less than primary school, *n* (%)	130 (59.1%)	135 (61.4%)	1.000
Current smoking, *n* (%)	20 (9.1%)	25 (11.4%)	0.561
History of diabetes, *n* (%)	16 (7.3%)	12 (5.5%)	0.558
History of CKD, *n* (%)	0 (0.0%)	2 (0.9%)	0.499
Mean TC, mmol/L (SD)	4.91 (0.8)	5.06 (1.0)	0.359
Mean TG, mmol/L (SD)	2.02 (1.7)	1.96 (1.6)	0.759
Mean LDL‐C, mmol/L (SD)	2.55 (0.7)	2.61 (0.8)	0.521
Mean HDL‐C, mmol/L (SD)	1.48 (0.3)	1.53 (0.4)	0.165
Mean plasma glucose, mmol/L (SD)	5.98 (1.8)	6.30 (2.1)	0.291
Mean ALT, U/L (SD)	18.46 (9.5)	19.33 (12.0)	0.275
Mean AST, U/L (SD)	20.79 (7.4)	21.66 (10.5)	0.749
Mean total protein, g/L (SD)	72.87 (5.2)	72.87 (5.2)	0.374
Mean urid acid, µmol/L (SD)	294.1 (85.0)	283.6 (75.4)	0.017
Mean serum creatinine, mg/dl (SD)	0.92 (0.2)	0.93 (0.2)	0.602
Mean BUN, mmol/L (SD)	5.55 (1.4)	5.72 (1.6)	0.464
Mean anti‐hypertensive medications (SD)	1.38 (1.3)	1.37 (1.4)	0.718
Mean SBP, mmHg (SD)	160.3 (18.0)	162.4 (20.4)	0.867
Mean DBP, mmHg (SD)	83.55 (10.0)	84.89 (11.5)	0.860
Mean PP, mmHg (SD)	76.79 (15.8)	77.50 (17.8)	0.511
Mean pTau217, pg/mL (SEM)	0.16 (0.02)	0.22 (0.02)	0.012

*Note*: This table presents baseline characteristics of participants. Values are reported as mean (SD) for continuous variables and *n* (%) for categorical variables. Group comparisons were performed using independent samples t tests for continuous variables and chi‐square or Fisher's exact tests for categorical variables. *P* values indicate overall group differences.

Abbreviations: ALT, alanine aminotransferase; AST, aspartate aminotransferase; BMI, body mass index; BUN, blood urea nitrogen; DBP, diastolic blood pressure HDL‐C, high‐density lipoprotein cholesterol; LDL‐C, low‐density lipoprotein cholesterol; SBP, systolic blood pressure; SD, standard deviation; TC, total cholesterol; TG, triglyceride.

### 3.4 Construction and discriminative performance of pTau217‐lipid composite indices

**FIGURE 3:**
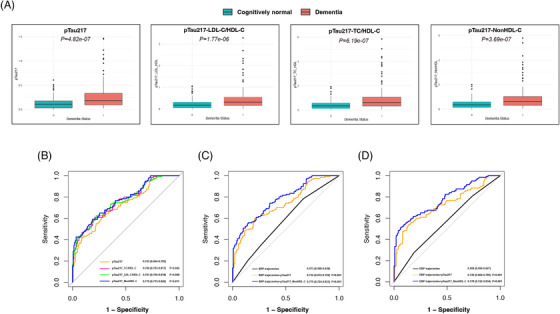
Distribution and discriminative performance of pTau217‐based composite indices and BP trajectories for dementia: nested case‐control study. (A) Distributions of serum pTau217 and pTau217‐lipid composite indices in participants with dementia and cognitively normal controls. Differences between groups were assessed using paired t tests for normally distributed variables or signed rank‐sum tests for non‐normally distributed variables. (B) ROC curves comparing the discriminative performance of pTau217 and the pTau217‐lipid composite indices for distinguishing dementia cases from cognitively normal controls. (C–D) ROC curves evaluating the incremental discriminative value of pTau217 and the optimal pTau217‐based composite index (pTau217‐NonHDL‐C) when added to systolic (C) and diastolic (D) BP trajectories. Areas under the curve with corresponding 95% confidence intervals were estimated using the DeLong test. BP, blood pressure; HDL‐C, high‐density lipoprotein cholesterol; ROC, receiver operating characteristic.

Next, we constructed three composite indices using baseline biomarkers, including pTau217 and lipid profiles (TC, LDL‐C, and HDL‐C). Regression analyses demonstrated significant interaction effects between pTau217 and selected lipid metabolism indices. Specifically, lipid metabolic dysfunction was found to modify the association between tau‐related neurodegeneration and dementia (**Table** **S8**). Among these indices, pTau217, pTau217‐LDL‐C/HDL‐C, pTau217‐TC/HDL‐C, and pTau217‐NonHDL‐C were significantly elevated in individuals with dementia compared with cognitively normal controls (**Figure** **3A**). ROC analyses were conducted to compare the discriminative performance of pTau217 alone with three pTau217‐based composite indices. Among these markers, pTau217‐NonHDL‐C demonstrated the highest discriminative ability for distinguishing dementia from cognitively normal controls (**Figure** **3B**) and was therefore selected for subsequent model comparisons. Using BP trajectory class assignments derived from the full cohort, ROC analyses showed that incorporating either pTau217 or pTau217‐composite indices substantially improved discriminative performance compared with BP trajectories alone. Notably, the addition of pTau217‐NonHDL‐C provided the greatest improvement in discrimination in models incorporating both SBP (**Figure** **3C**) and DBP trajectories (**Figure** **3D**). Sensitivity analyses based on BP trajectories re‐estimated within the nested case‐control sample produced nearly identical results (**Figure** **S2**).

### 3.5 pTau217‐lipid composite indices modify BP trajectory‐dementia associations

**FIGURE 4:**
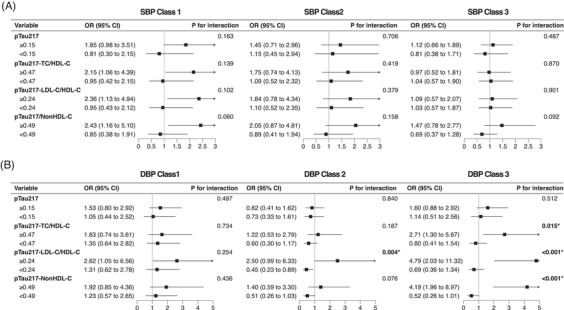
Interaction between baseline pTau217 and composite indices with BP trajectories on dementia: nested case‐control study. ORs and 95% CIs for the interactions between SBP and DBP trajectories and baseline pTau217 and composite indices were estimated using generalized linear mixed models with random intercepts for village (cluster) and matched pair. Data are presented for BP trajectories classes 1–3, with class 4 as the reference. *P* values for interactions are shown for each model. CI, confidence interval; OR, odds ratio.

Given the observed associations between BP trajectories and dementia, we further evaluated potential effect modification by baseline neurodegenerative and metabolic vulnerability biomarkers. In the full cohort, no statistically significant effect modification by age or sex was observed for either SBP or DBP trajectories (**Figures** **Figures** S3 and S4). In the nested case–control analysis, BP trajectory class assignments derived from the full cohort were applied. For SBP trajectories, no statistically significant interaction with pTau217‐lipid composite indices was observed in the primary analysis, although a borderline interaction was noted for SBP class 1 with pTau217‐NonHDL‐C (*P* for interaction = 0.060). In contrast, for DBP trajectories, associations with dementia were more pronounced among participants with elevated pTau217‐lipid composite indices. Specifically, compared with class 4, stronger associations were observed in class 2 among participants with pTau217‐LDL‐C/HDL‐C ≥ 0.24, and in class 3 among participants with pTau217‐TC/HDL‐C ≥ 0.47, pTau217‐LDL‐C/HDL‐C ≥ 0.24, and pTau217‐NonHDL‐C ≥ 0.49 (**Figure** **4**). Among the examined stratification factors, pTau217‐lipid composite indices provided the most consistent evidence suggestive of effect modification across DBP trajectories. Sensitivity analyses based on BP trajectories re‐estimated within the nested case‐control sample yielded nearly identical results (**Figure** **S5**).

## 4 DISCUSSION

This study provides new insights into the associations between long‐term BP trajectories and dementia in a hypertensive population, highlighting the modifying roles of neurodegenerative and metabolic factors. We found that the association between BP trajectories and dementia was most pronounced among individuals with high‐stable or high‐declining systolic and diastolic BP trajectories. Individuals with higher serum pTau217‐lipid composite indices showed the most pronounced effect modification across DBP trajectories. Together, these findings indicate that integrating BP trajectory monitoring with pTau217 and lipid metabolic assessment offers a more refined framework for dementia stratification and personalized prevention in old hypertensive adults.

### 4.1 BP trajectories and dementia in hypertensive populations

Previous studies examining BP trajectories in relation to dementia have largely been conducted in broadly defined community populations.^8^, ^9^, ^10^, ^12^, ^41^ In these studies, trajectories associated with dementia typically involved a gradual decline in systolic and diastolic BP preceding dementia onset, or an initial increase followed by subsequent decline, with most reports focusing primarily on systolic BP patterns. However, subgroup analyses from several cohorts have suggested that BP trajectories associated with dementia among individuals with established hypertension differ from those observed in the general population,^8^, ^13^ indicating that baseline hypertensive status may fundamentally alter the relationship between BP dynamics and dementia.

To date, few studies have specifically focused on BP trajectories and dementia within exclusively hypertensive populations. Leveraging the CRHCP cohort of 28,135 individuals with hypertension and five BP assessments over a 4‐year period, we identified four distinct BP trajectory patterns. In fully adjusted analyses, the high‐stable DBP trajectory showed the strongest association with dementia, followed by the high‐stable SBP trajectory, the high‐declining SBP trajectory, the moderate‐stable DBP trajectory, and the high‐declining DBP trajectory. Notably, this pattern aligns with findings from a prior population‐based cohort of 10,660 participants followed for 10 years,^13^ in which persistently high DBP trajectory was associated with the dementia among individuals diagnosed with hypertension at baseline. These findings highlight the potential importance of DBP trajectories in dementia among hypertensive individuals, a relationship that may be less apparent in mixed or normotensive populations.

As for SBP, previous evidence from late‐life cohorts indicates a threshold‐dependent association between SBP and dementia in hypertensive older adults. Studies in individuals aged 65–74 years have reported that maintaining SBP within 140–159 mmHg up to 6 years before dementia diagnosis was not associated with dementia, whereas persistently high SBP ≥160 mmHg was linked to dementia.^15^ Consistent with these findings, in our study, using class 4 (moderate‐declining) as the reference group, the moderate‐stable SBP trajectory (class 3; mean baseline SBP = 148.6 mmHg) was not associated with dementia in the overall analysis, whereas the high‐stable trajectory (class 1; mean baseline SBP = 171.6 mmHg) showed a significant association. Together, these results support a threshold‐dependent relationship between sustained SBP elevation and dementia among individuals with established hypertension.

Regarding the overall BP trajectory patterns, we observed a decline in both SBP and DBP in several trajectories during follow‐up. Substantial evidence indicates that both SBP and DBP tend to decrease in later life.^42^, ^43^, ^44^ These trends may reflect a combination of age‐related reductions in vascular elasticity, progressive renal changes, and metabolic alterations.^45^ Even among hypertensive individuals, declines in both SBP and DBP are commonly observed in later life.^46^ Additionally, several studies have reported that BP often begins to decline approximately 3–5 years before dementia onset,^10^, ^11^, ^15^ suggesting that such decline may coincide with early neurodegenerative processes.

### 4.2 Clinical and demographic heterogeneity across BP trajectory patterns

The relationship between BP trajectories and dementia is influenced by factors such as age, sex, and cardiovascular disease background. Existing evidence on the association between BP trajectories and dementia across different age groups has been inconsistent. Some studies have shown a significant association between declining SBP and dementia, but this was observed only in individuals aged ≥80 years, with no such relationship seen in those aged 60–79 years.^13^ Similarly, a declining SBP trajectory was linked to dementia only in patients aged ≥78 years, while elevated SBP was associated with dementia in those aged < 78 years.^11^ However, another longitudinal study suggested that a declining BP trajectory in late life was not associated with dementia in individuals aged ≥74 years but was linked to dementia in those aged < 74 years (the age at cognitive assessment).^47^ In terms of sex differences, previous studies suggest that compared to men, women exhibit a stronger association between BP levels and dementia.^48^ In our hypertensive population‐based full cohort, we did not observe statistically significant effect modification by age or sex for BP trajectories. This finding is consistent with evidence from large‐scale pooled analyses incorporating data from the Health and Retirement Study (HRS),^49^ the English Longitudinal Study of Ageing (ELSA),^50^ and the China Health and Retirement Longitudinal Study (CHARLS),^51^ which reported no statistically significant interaction between age or sex and BP variability in relation to dementia.^52^

### 4.3 Interaction of pTau217‐lipid composite indices with BP trajectories for dementia

Dementia arises from the interplay of vascular dysfunction, neurodegeneration, and metabolic abnormalities.^53^, ^54^ In line with this framework, lipid metabolic disturbances have been consistently associated with dementia,^19^ and our interaction analyses further demonstrated that individuals with baseline lipid abnormalities exhibited stronger associations between BP trajectories and dementia. Notably, composite indices integrating pTau217 with lipid‐related markers showed stronger associations than any single biomarker alone, supporting their role as indicators of underlying biological vulnerability.

Within the nested case‐control sample, DBP class 3 (moderate‐stable) and class 2 (high‐declining) exhibited the pronounced differentiation in dementia across pTau217‐lipid composite indices. The stronger association observed for DBP class 3 was predominantly evident among individuals with elevated pTau217‐lipid composite indices, suggesting that this trajectory pattern may confer greater dementia vulnerability in the presence of underlying biological susceptibility. However, SBP class 1 and DBP class 1 (high‐stable) were consistently associated with dementia regardless of baseline pTau217‐lipid composite indices levels, suggesting that persistently high BP may be associated with dementia independently of underlying biological vulnerability.

In the full cohort analysis, relative to class 4, DBP class 3 exhibited a stronger association with dementia than DBP class 2. Inspection of trajectory morphology revealed that DBP class 2 was characterized by a marked decline from a higher baseline to normal levels (baseline mean DBP = 94.43 mmHg; final mean = 72.49 mmHg). In contrast, DBP class 3 exhibited persistently high‐normal diastolic levels throughout follow‐up (baseline mean = 83.93 mmHg; final mean = 80.77 mmHg), suggesting sustained microvascular stress. Prolonged exposure to high‐normal DBP may contribute to cerebral small‐vessel dysfunction and hypoperfusion, processes implicated in amyloid deposition and tau‐related neurodegeneration.^55^, ^56^ Consistent with our findings, prior studies have reported that moderate‐stable DBP trajectories are more strongly associated with dementia than trajectories characterized by pronounced DBP decline to normotensive levels.^5^, ^13^

Collectively, these findings support a framework that integrates longitudinal BP trajectories with biological vulnerability, rather than relying solely on single BP thresholds, thereby advancing a more individualized approach to dementia stratification in hypertensive older adults.

### 4.4 Limitations

There are several limitations to our study. First, despite statistical adjustment for randomization arm and village‐level clustering, residual confounding related to unobserved covariates cannot be entirely excluded in the analysis of observational data. Treatment‐related behavioral modifications and medication adherence may have influenced longitudinal BP dynamics. Therefore, the findings should be interpreted as associative rather than causal. Second, although the full cohort was large, the nested case–control sample was modest in size, which limited statistical power for subgroup analyses. Third, the relatively short follow‐up period may have limited our ability to detect late‐onset dementia or fully evaluate the long‐term effects of BP trajectories on dementia development. Because dementia outcomes were assessed at a single visit, it is possible that, for some participants, BP trajectory assessment partially overlapped with the preclinical or early clinical phase of dementia. This temporal overlap should be considered when interpreting the observed associations. Fourth, while serum pTau217 has shown promise as a biomarker for Alzheimer's pathology, its sensitivity and specificity in detecting early all‐cause dementia are still under evaluation. Finally, Tau phosphorylation may begin many years before the clinical onset of dementia. Therefore, some participants with elevated baseline pTau217 levels may already have been in a preclinical stage at study entry, which may have influenced the observed associations.

### 4.5 Implications for research

Routine‐based BP measurement may not fully capture the longitudinal patterns that relate to dementia. Incorporating repeated BP assessments, either from clinic visits or home monitoring, may improve dementia stratification frameworks. From a research perspective, BP trajectory monitoring could be evaluated alongside serum pTau217 and lipid metabolic markers within existing cognitive assessment settings. This combined approach would help identify individuals whose specific BP patterns confer high dementia risk, thereby prioritizing them for tailored interventions, including vascular risk control, lifestyle modification, and closer cognitive surveillance. In our study, hypertensive individuals with high‐stable SBP or DBP trajectories showed consistent associations with dementia irrespective of baseline biomarker levels. In contrast, for high‐declining or moderate‐stable trajectories, elevated pTau217‐lipid composite indices were associated with stronger associations with dementia. These findings support a BP trajectory‐aware, biomarker‐informed framework for dementia management.

## 5 CONCLUSIONS

High‐stable systolic and diastolic BP trajectories were consistently associated with dementia, whereas the associations of high‐declining and moderate‐stable trajectories varied according to pTau217‐lipid composite indices.

## CONFLICT OF INTEREST STATEMENT

The authors declare no conflicts of interest. Author disclosures are available in the supporting information.

## CONSENT STATEMENT

The CRHCP study was approved by the institutional review board at each participating site, and each participant provided written informed consent.

## Supporting information



Supporting Information

Supporting Information

## Data Availability

The data and codes analyzed during the current study are available from the corresponding author on reasonable request.
