# Supplementary material for: Longitudinal blood pressure trajectories and tau‐lipid biomarkers associated with dementia in hypertensive adults: Results from the population‐based CRHCP cohort
Source: Alzheimers Dement. 2026 Apr 6;22(4):e71346. doi: 10.1002/alz.71346 (PMC13053948; doi:10.1002/alz.71346)
Supplement: Supplementary file 2 — Supporting Information [file ALZ-22-e71346-s001.pdf]

Table S1. Fit indices for two- to six-class growth mixture models for SBP

Classes	Loglikelihood	AIC	BIC	SSA-BIC	LMR-test	BLRT	Entropy
**2-class**	-493698.845	974195.275	974323.584	974272.736	<0.0001	<0.0001	0.752
**3-class**	-487081.638	971768.530	971944.954	971875.039	<0.0001	<0.0001	0.735
**4-class**	**-485862.265**	**969414.424**	**969638.964**	**969549.981**	**<0.0001**	**<0.0001**	**0.773**
**5-class**	-483939.740	967947.479	968220.134	968112.084	<0.0001	<0.0001	0.771
**6-class**	-483456.827	966993.653	967314.424	967187.305	<0.0001	<0.0001	0.755

AIC, Akaike information criterion; BIC, Bayesian information criterion; SSA-BIC, Sample-size adjusted BIC; LMR-test, Lo-Mendell-Rubin adjusted likelihood ratio test; BLRT, bootstrap likelihood ratio test.

Table S2. Fit indices for two- to six-class growth mixture models for DBP

Classes	Loglikelihood	AIC	BIC	SSA-BIC	LMR-test	BLRT	Entropy
**2-class**	-431172.094	847450.519	847578.827	847527.980	<0.0001	<0.0001	0.656
**3-class**	-423709.260	842899.557	843075.981	843006.066	<0.0001	<0.0001	0.700
**4-class**	**-421427.779**	**841723.517**	**841948.056**	**841859.073**	**<0.0001**	**<0.0001**	**0.726**
**5-class**	-420833.758	840409.021	840681.676	840573.625	<0.0001	<0.0001	0.701
**6-class**	-420170.510	839595.760	839916.530	839789.412	<0.0001	<0.0001	0.719

AIC, Akaike information criterion; BIC, Bayesian information criterion; SSA-BIC, Sample-size adjusted BIC; LMR-test, Lo-Mendell-Rubin adjusted likelihood ratio test; BLRT, bootstrap likelihood ratio test.

Table S3. Fit indices for two- to six-class growth mixture models for PP

Classes	Loglikelihood	AIC	BIC	SSA-BIC	LMR-test	BLRT	Entropy
**2-class**	-469270.395	920172.314	920300.622	920249.775	<0.0001	<0.0001	0.795
**3-class**	-460070.157	915864.633	916041.057	915971.142	<0.0001	<0.0001	0.719
**4-class**	**-457910.317**	**913370.695**	**913595.234**	**913506.251**	**<0.0001**	**<0.0001**	**0.755**
**5-class**	-456657.347	911859.427	912132.082	912024.031	0.0015	0.0016	0.759
**6-class**	-455895.713	910305.798	910626.569	910499.450	0.0148	0.0156	0.742

AIC, Akaike information criterion; BIC, Bayesian information criterion; SSA-BIC, Sample-size adjusted BIC; LMR-test, Lo-Mendell-Rubin adjusted likelihood ratio test; BLRT, bootstrap likelihood ratio test.

Table S4. Baseline demographics of the full cohort across SBP trajectories

Variables	Class 1	Class 2	Class 3	Class 4
**Total, n (%)**	3584 (12.74%)	5211 (18.52%)	7895 (28.06%)	11445 (40.68%)
**Dementia, n (%)**	277 (7.73%)	321 (6.16%)	381 (4.83%)	423 (3.70%)
**Female, n (%)**	2261 (63.09%)	3304 (63.40%)	4760 (60.29%)	7076 (61.83%)
**Age, median years (IQR)**	70 (64-75)	69 (62-74)	68 (60-73)	66 (59-72)
**Education, n (%)**				
<6 years	2670 (74.50%)	3700 (71.00%)	5355 (67.83%)	7416 (64.80%)
6-9 years	753 (21.01%)	1290 (24.76%)	2123 (26.89%)	3350 (29.27%)
>9 years	161 (4.49%)	221 (4.24%)	417 (5.28%)	679 (5.93%)
**BMI (kg/m^2), mean (SD)**	26.07 (4.11)	26.26 (3.89)	25.92 (3.73)	26.02 (3.70)
**Physical activity level†**				
Active	1228 (34.26%)	2196 (42.14%)	3039 (38.49%)	5085 (44.43%)
Insufficiently active	1189 (33.18%)	1624 (31.16%)	2581 (32.69%)	2896 (25.30%)
Inactive	1167 (32.56%)	1391 (26.70%)	2275 (28.82%)	3464 (30.27%)
**Smoking status, n (%)**				
Current	631 (17.61%)	864 (16.58%)	1529 (19.37%)	2133 (18.64%)
Former	276 (7.70%)	357 (6.85%)	525 (6.65%)	763 (6.67%)
Never	2677 (74.69%)	3990 (76.57%)	5841 (73.98%)	8549 (74.70%)
**Alcohol, n (%)**				
Current	495 (13.81%)	696 (13.36%)	1130 (14.31%)	1701 (14.86%)
Former	246 (6.86%)	335 (6.43%)	453 (5.74%)	738 (6.45%)
Never	2843 (79.32%)	4180 (80.21%)	6312 (79.95%)	9006 (78.69%)
**Diabetes, n (%)**	293 (8.18%)	461 (8.85%)	675 (8.55%)	977 (8.54%)
**CKD, n (%)**	17 (0.47%)	27 (0.52%)	48 (0.61%)	67 (0.59%)
**AF, n (%)**	29 (0.81%)	32 (0.61%)	69 (0.87%)	88 (0.77%)
**MI, n (%)**	89 (2.48%)	137 (2.63%)	185 (2.34%)	301 (2.63%)
**Angina, n (%)**	135 (3.77%)	201 (3.86%)	319 (4.04%)	476 (4.16%)
**Stroke, n (%)**	1157 (32.28%)	1606 (30.82%)	2247 (28.46%)	3052 (26.67%)
**Medications, mean (SD)**	1.16 (1.09)	2.73 (1.49)	1.14 (1.16)	2.43 (1.41)
**Glucose (mmol/L), mean (SD)**	6.28 (2.24)	6.26 (1.96)	6.11 (1.93)	6.10 (1.96)
**TG (mmol/L), mean (SD)**	2.04 (1.69)	2.02 (1.69)	1.94 (1.57)	1.96 (1.63)
**TC (mmol/L), mean (SD)**	5.14 (1.03)	5.12 (1.01)	5.01 (1.00)	5.00 (1.01)
**LDL-C (mmol/L), mean (SD)**	2.76 (0.85)	2.74 (0.83)	2.70 (0.81)	2.68 (0.82)
**HDL-C (mmol/L), mean (SD)**	1.45 (0.34)	1.46 (0.35)	1.43 (0.35)	1.44 (0.34)
**Total protein (g/L), mean (SD)**	74.31 (5.50)	74.38 (5.01)	73.69 (5.18)	73.58 (4.87)

**TBil (µmol/L), mean (SD)**	11.87 (5.86)	11.86 (5.66)	11.90 (5.62)	12.11 (5.63)
**ALT (U/L), mean (SD)**	21.54 (15.67)	22.21 (16.18)	21.81 (16.39)	22.43 (15.27)
**AST (U/L), mean (SD)**	22.55 (11.61)	22.27 (11.75)	22.08 (12.07)	21.94 (10.21)
**Cr (mg/dL), mean (SD)**	0.91 (0.20)	0.91 (0.19)	0.89 (0.19)	0.90 (0.19)
**Urid acid (µmol/L), mean (SD)**	306.7 (85.18)	299.9 (83.11)	303.7 (85.28)	299.7 (86.15)
**BUN (µmol/L), mean (SD)**	5.50 (1.55)	5.45 (1.51)	5.50 (1.54)	5.45 (2.63)

BMI, body mass index; CKD: chronic kidney disease; AF: atrial fibrillation; MI: myocardial infarction; Glu, glucose; LDL-C, low-density lipoprotein cholesterol; HDL-C, high-density lipoprotein cholesterol; TC, total cholesterol; TG, triglycerides; Cr, creatinine; BUN, blood urea nitrogen.

†Physical activities were then classified into three activity levels using the current adult aerobic guideline:

1) active, reporting at least 150 minutes/week of moderate-intensity equivalent physical activity.

2) insufficiently active, reporting some moderate-intensity equivalent physical activity but not enough to meet active definition.

3) inactive, reporting no moderate-intensity equivalent physical activity that lasted at least 10 minutes.

Table S5. Baseline demographics of the full cohort across DBP trajectories

Variables	Class 1	Class 2	Class 3	Class 4
**Total, n (%)**	5121 (18.20%)	7835 (27.85%)	7258 (25.80%)	7921 (28.15%)
**Dementia, n (%)**	262 (5.12%)	300 (3.83%)	393 (5.41%)	447 (5.64%)
**Female, n (%)**	2801 (54.70%)	4565 (58.26%)	4648 (64.04%)	5534 (69.86%)
**Age, median years (IQR)**	64 (57-70)	65 (58-71)	68 (62-74)	71 (66-76)
**Education, n (%)**				
<6 years	3229 (63.05%)	4996 (63.77%)	5046 (69.52%)	5870 (74.11%)
6-9 years	1551 (30.29%)	2388 (30.48%)	1846 (25.43%)	1731 (21.85%)
>9 years	341 (6.66%)	451 (5.76%)	366 (5.04%)	320 (4.04%)
**BMI (kg/m^2), mean (SD)**	26.64 (3.95)	26.41 (3.77)	25.84 (3.73)	25.48 (3.71)
**Physical activity level†**				
Active	2042 (39.88%)	3489 (44.53%)	2855 (39.34%)	3162 (39.92%)
Insufficiently active	1449 (28.30%)	1950 (24.89%)	2097 (28.89%)	2233 (28.19%)
Inactive	1630 (31.83%)	2396 (30.58%)	2306 (31.77%)	2526 (31.89%)
**Smoking status, n (%)**				
Current	1106 (21.60%)	1601 (20.43%)	1268 (17.47%)	1182 (14.92%)
Former	397 (7.75%)	561 (7.16%)	485 (6.68%)	478 (6.03%)
Never	3618 (70.65%)	5673 (72.41%)	5505 (75.85%)	6261 (79.04%)
**Alcohol, n (%)**				
Current	923 (18.02%)	1308 (16.69%)	969 (13.35%)	822 (10.38%)
Former	343 (6.70%)	561 (7.16%)	443 (6.10%)	425 (5.37%)
Never	3855 (75.28%)	5966 (76.15%)	5846 (80.55%)	6674 (84.26%)
**Diabetes, n (%)**	247 (4.82%)	554 (7.07%)	656 (9.04%)	949 (11.98%)
**CKD, n (%)**	31 (0.61%)	40 (0.51%)	34 (0.47%)	54 (0.68%)
**AF, n (%)**	37 (0.72%)	38 (0.49%)	73 (1.01%)	70 (0.88%)
**MI, n (%)**	99 (1.93%)	170 (2.17%)	207 (2.85%)	236 (2.98%)
**Angina, n (%)**	162 (3.16%)	277 (3.54%)	317 (4.37%)	375 (4.73%)
**Stroke, n (%)**	1506 (29.41%)	2160 (27.57%)	2084 (28.71%)	2312 (29.19%)
**Medications, mean (SD)**	1.26 (1.25)	2.52 (1.46)	1.43 (1.33)	2.35 (1.47)
**Glucose (mmol/L), mean (SD)**	5.99 (1.81)	6.10 (1.88)	6.15 (2.02)	6.30 (2.17)
**TG (mmol/L), mean (SD)**	2.09 (1.83)	2.05 (1.71)	1.90 (1.52)	1.91 (1.51)
**TC (mmol/L), mean (SD)**	5.02 (1.01)	5.05 (1.01)	5.03 (1.01)	5.06 (1.02)
**LDL-C (mmol/L), mean (SD)**	2.68 (0.81)	2.69 (0.81)	2.71 (0.82)	2.74 (0.84)
**HDL-C (mmol/L), mean (SD)**	1.42 (0.35)	1.44 (0.35)	1.45 (0.34)	1.45 (0.34)
**Total protein (g/L), mean (SD)**	73.97 (5.29)	73.98 (4.96)	73.68 (5.14)	73.80 (5.00)

**TBil (μmol/L), mean (SD)**	12.31 (5.92)	12.29 (5.69)	11.82 (5.61)	11.59 (5.48)
**ALT (U/L), mean (SD)**	22.90 (15.18)	23.32 (20.15)	21.34 (14.06)	21.01 (13.00)
**AST (U/L), mean (SD)**	22.48 (11.94)	22.48 (13.81)	21.80 (9.31)	21.82 (9.35)
**Cr (mg/dL), mean (SD)**	0.90 (0.19)	0.90 (0.19)	0.90 (0.19)	0.90 (0.19)
**Urid acid (μmol/L), mean (SD)**	313.2 (88.45)	305.3 (88.80)	297.7 (82.28)	294.4 (81.20)
**BUN (μmol/L), mean (SD)**	5.37 (1.51)	5.37 (1.50)	5.55 (3.08)	5.57 (1.57)

BMI, body mass index; CKD: chronic kidney disease; AF: atrial fibrillation; MI: myocardial infarction; Glu, glucose; LDL-C, low-density lipoprotein cholesterol; HDL-C, high-density lipoprotein cholesterol; TC, total cholesterol; TG, triglycerides; Cr, creatinine; BUN, blood urea nitrogen.

†Physical activities were then classified into three activity levels using the current adult aerobic guideline:

1) active, reporting at least 150 minutes/week of moderate-intensity equivalent physical activity.

2) insufficiently active, reporting some moderate-intensity equivalent physical activity but not enough to meet active definition.

3) inactive, reporting no moderate-intensity equivalent physical activity that lasted at least 10 minutes.

Table S6. Baseline characteristics by SBP trajectories: nested case-control study

Variables	Class 1	Class 2	Class 3	Class 4	P value
**Total, n (%)**	90 (20.45%)	61 (13.86%)	176 (40.00%)	113 (25.68%)	
**Age (years)**	74.38 ± 6.93	76.19 ± 6.49	76.83 ± 7.40	75.16 ± 7.24	0.157
**BMI (kg/m²)**	25.02 ± 4.34	24.55 ± 3.46	25.01 ± 3.69	24.86 ± 3.61	0.922
**Female, n (%)**	84 (93.3%)	50 (82.0%)	130 (73.9%)	95 (84.1%)	**0.018***
**Education, n (%)**					0.174
**>6 years**	37 (41.1%)	17 (27.9%)	72 (40.9%)	40 (35.4%)	
**<6 years**	53 (58.9%)	44 (72.1%)	104 (59.1%)	73 (64.6%)	
**Diabetes, n (%)**	0 (0.0%)	2 (3.3%)	20 (11.4%)	5 (4.4%)	**0.012***
**TC (mmol/L)**	5.09 ± 0.97	4.86 ± 0.84	4.90 ± 0.85	5.04 ± 1.05	0.515
**TG (mmol/L)**	2.12 ± 2.37	2.25 ± 1.43	1.85 ± 1.30	2.01 ± 1.81	0.591
**LDL-C (mmol/L)**	2.65 ± 0.80	2.44 ± 0.68	2.55 ± 0.73	2.63 ± 0.80	0.513
**HDL-C (mmol/L)**	1.54 ± 0.35	1.47 ± 0.27	1.49 ± 0.35	1.50 ± 0.34	0.788
**Glucose (mmol/L)**	6.08 ± 2.40	6.33 ± 1.36	6.39 ± 2.40	5.77 ± 1.24	0.213
**Cr (mg/dL)**	0.90 ± 0.18	0.97 ± 0.15	0.94 ± 0.15	0.90 ± 0.17	**0.042***
**ALT (U/L)**	18.91 ± 14.65	16.53 ± 5.06	19.33 ± 11.43	19.68 ± 9.02	0.531
**AST (U/L)**	21.47 ± 8.36	19.80 ± 4.87	21.97 ± 12.02	20.67 ± 6.33	0.586
**Total protein (g/L)**	74.64 ± 5.70	73.11 ± 5.13	72.12 ± 5.36	72.65 ± 4.41	0.051
**Urid acid (μmol/L)**	284.1 ± 89.10	293.0 ± 75.72	297.4 ± 84.64	275.1 ± 72.07	0.317
**BUN (μmol/L)**	5.60 ± 1.50	5.90 ± 1.69	5.69 ± 1.48	5.43 ± 1.42	0.436
**Anti-hypertensive medications**	1.02 ± 0.78	2.11 ± 1.49	0.77 ± 0.91	2.13 ± 1.51	**<0.001***
**Baseline SBP (mmHg)**	177.1 ± 17.19	186.0 ± 15.49	153.2 ± 10.51	149.7 ± 11.16	**<0.001***
**Baseline DBP (mmHg)**	88.53 ± 12.47	90.84 ± 9.61	80.70 ± 9.47	83.37 ± 9.38	**<0.001***
**Baseline PP (mmHg)**	89.17 ± 17.69	95.11 ± 15.09	72.47 ± 9.99	66.32 ± 11.25	**<0.001***
**Baseline pTau217 (pg/mL)**	0.17 ± 0.03	0.24 ± 0.04	0.19 ± 0.02	0.20 ± 0.03	0.224

Note: This table presents baseline characteristics of the study population stratified by SBP trajectories. Values are reported as mean (SD) for continuous variables and n (%) for categorical variables. Group differences across SBP classes were tested using analysis of variance (ANOVA) for continuous variables and chi-square tests for categorical variables.

Table S7. Baseline characteristics by DBP trajectories: nested case-control study

Variables	Class 1	Class 2	Class 3	Class 4	P value
**Total, n (%)**	87 (19.77%)	141 (32.05%)	125 (28.41%)	87 (19.77%)	
**Age (years)**	74.45 ± 6.80	75.56 ± 7.76	75.68 ± 7.05	77.81 ± 6.49	0.095
**BMI (kg/m²)**	25.33 ± 4.36	25.05 ± 3.52	24.66 ± 3.31	24.54 ± 3.76	0.605
**Female, n (%)**	67 (77.0%)	115 (81.6%)	96 (76.8%)	81 (93.1%)	0.075
**Education, n (%)**					
**>6 years**	38 (43.7%)	52 (36.9%)	46 (36.8%)	29 (33.3%)	0.813
**<6 years**	49 (56.3%)	89 (63.1%)	79 (63.2%)	58 (66.7%)	
**Diabetes, n (%)**	2 (2.3%)	11 (7.8%)	9 (7.2%)	5 (5.7%)	0.460
**TC (mmol/L)**	4.88 ± 1.04	4.99 ± 0.81	5.07 ± 0.93	4.93 ± 0.93	0.646
**TG (mmol/L)**	2.11 ± 2.13	1.84 ± 1.29	1.90 ± 1.40	2.15 ± 1.83	0.657
**LDL-C (mmol/L)**	2.48 ± 0.75	2.57 ± 0.70	2.62 ± 0.82	2.63 ± 0.73	0.644
**HDL-C (mmol/L)**	1.49 ± 0.33	1.52 ± 0.33	1.55 ± 0.36	1.44 ± 0.33	0.222
**Glucose (mmol/L)**	5.83 ± 1.13	6.28 ± 2.60	6.09 ± 1.80	6.31 ± 2.22	0.521
**Cr (mg/dL)**	0.92 ± 0.17	0.95 ± 0.17	0.92 ± 0.14	0.91 ± 0.17	0.624
**ALT (U/L)**	20.13 ± 13.03	18.72 ± 9.02	18.38 ± 11.79	18.31 ± 8.26	0.763
**AST (U/L)**	21.88 ± 7.85	20.67 ± 5.94	21.27 ± 12.18	21.16 ± 8.50	0.908
**Total protein (g/L)**	72.57 ± 5.13	73.19 ± 4.81	72.52 ± 5.84	73.54 ± 4.74	0.610
**Urid acid (µmol/L)**	281.60 ± 70.77	285.03 ± 80.68	295.88 ± 88.27	291.35 ± 79.50	0.726
**BUN (µmol/L)**	5.32 ± 1.31	5.96 ± 1.58	5.61 ± 1.48	5.65 ± 1.64	0.135
**Anti-hypertensive medications**	1.05 ± 1.21	1.83 ± 1.50	1.03 ± 1.05	1.75 ± 1.41	**<0.001***
**Baseline SBP (mmHg)**	168.1 ± 19.34	165.1 ± 20.33	155.0 ± 16.72	158.0 ± 17.16	**<0.001***
**Baseline DBP (mmHg)**	93.94 ± 8.95	90.45 ± 6.99	79.16 ± 6.29	74.63 ± 7.52	**<0.001***
**Baseline PP (mmHg)**	74.19 ± 14.82	74.64 ± 17.64	75.83 ± 15.80	83.35 ± 16.48	**0.005**
**Baseline pTau217 (pg/mL)**	0.14 ± 0.01	0.22 ± 0.04	0.19 ± 0.02	0.23 ± 0.03	0.124

Note: This table presents baseline characteristics of the study population stratified by DBP trajectory. Values are reported as mean (SD) for continuous variables and n (%) for categorical variables. Group differences across DBP classes were tested using analysis of variance (ANOVA) for continuous variables and chi-square tests for categorical variables.

Table S8. Multiple linear regression analysis for the interaction between pTau217 and lipid indices for dementia risk

	Coefficients				
**Dependent variable: Dementia**	**B**	**SE**	**β**	**t**	**P value**
pTau217	0.639	0.131	2.885	4.875	1.848e-06
TC/HDL-C	0.086	0.029	0.082	2.990	3.046e-03
LDL-C/HDL-C	0.112	0.041	0.154	2.751	6.333e-03
NonHDL-C	0.118	0.032	0.130	3.684	2.773e-04
pTau217 x TC/HDL-C	1.151	0.540	5.199	2.133	3.381e-02
pTau217 x LDL-C/HDL-C	1.011	0.468	4.565	2.160	3.169e-02
pTau217 x NonHDL-C	1.050	0.568	4.743	1.848	4.569e-02

Note: Multiple linear regression model included age, gender, education, and BMI as covariates. The table presents the coefficients (B), standard errors (SE), standardized coefficients (β), t-values, and p-values for the association between pTau217 deposition, lipid indices, and their interactions on dementia. Abbreviations: LDL-C, low density lipoprotein cholesterol; HDL-C, high density lipoprotein cholesterol; TC: total cholesterol.

Figure S1. Blood pressure trajectories: Sensitivity analysis in the nested case-control study

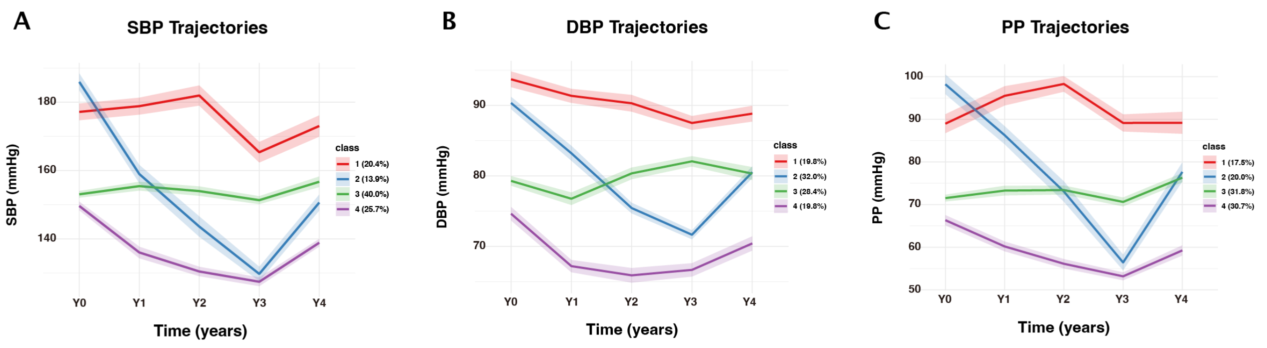

Re-identified trajectories of systolic blood pressure (SBP, A), diastolic blood pressure (DBP, B), and pulse pressure (PP, C) by latent class group-based models (LCGBM) in the nested case-control study. The x-axis represents time across five visits (Y0-Y4), and the y-axis shows the BP values for each class. (1) high-stable; (2) high-declining; (3) moderate-stable; (4) moderate-declining.

Figure S2. Discriminative performance of pTau217-based composite indices and BP trajectories for dementia: Sensitivity analysis in nested case-control study

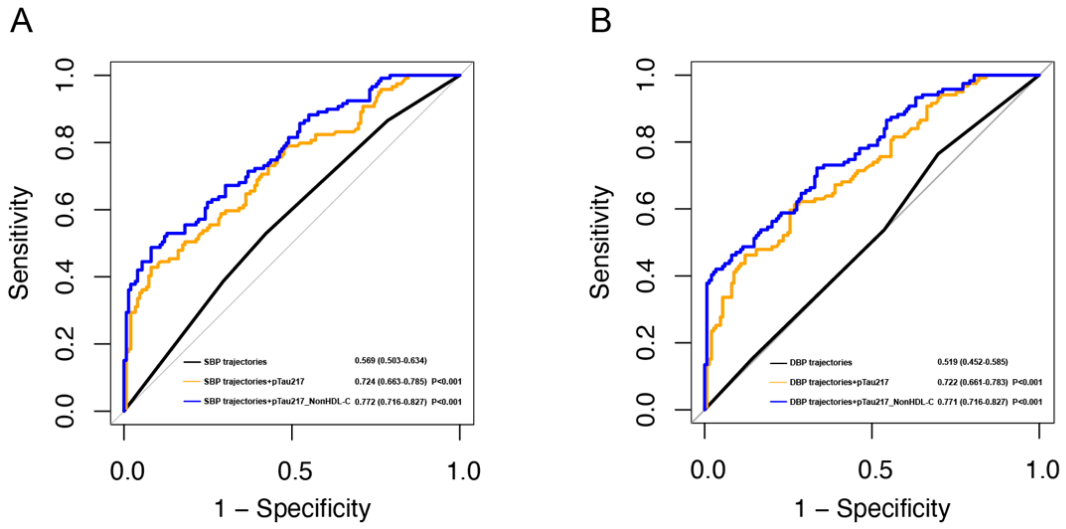

Note: ROC curves evaluating the incremental discriminative value of pTau217 and pTau217-NonHDL-C when added to systolic (A) and diastolic BP (B) trajectory patterns derived from the nested case-control study. Comparisons include SBP/DBP trajectories alone, SBP/DBP trajectories combined with pTau217, and SBP/DBP trajectories combined with pTau217-NonHDL-C. Areas under the curve (AUCs) with corresponding 95% confidence intervals were estimated using the DeLong's test.

Figure S3. Interaction between baseline characteristics with SBP trajectories on dementia risk: Full cohort study

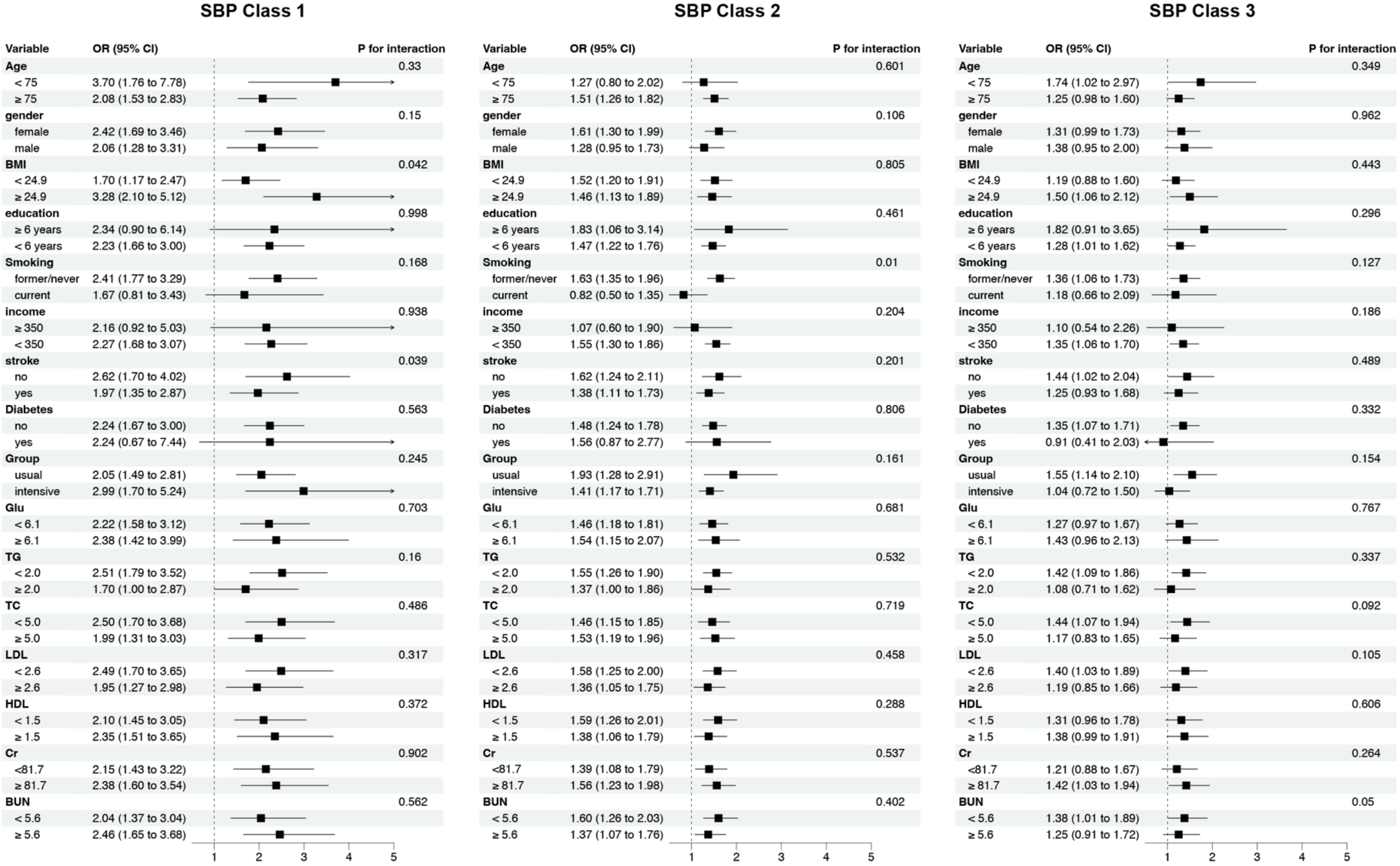

Note: Interaction odds ratios (ORs) and 95% confidence intervals (CIs) for the association between each SBP trajectory class and dementia, compared with the reference class (class 4). BMI, body mass index; CI, confidence interval; Glu, glucose; LDL-C, low-density lipoprotein cholesterol; HDL-C, high-density lipoprotein cholesterol; TC, total cholesterol; TG, triglycerides; Cr, creatinine; BUN, blood urea nitrogen.

Figure S4. Interaction between baseline characteristics with DBP trajectories on dementia risk: Full cohort study

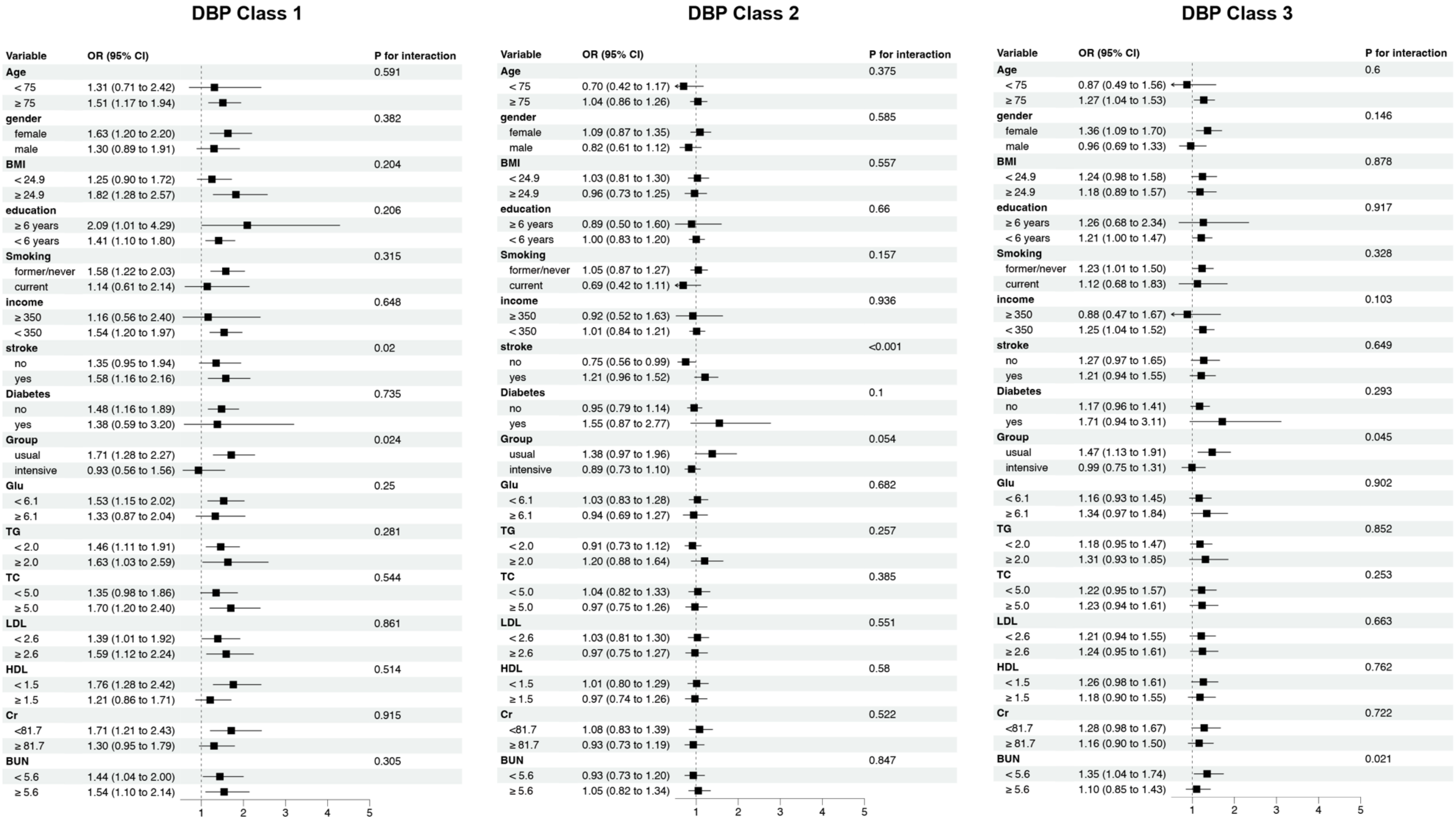

Note: Interaction odds ratios (ORs) and 95% confidence intervals (CIs) for the association between each DBP trajectory class and dementia, compared with the reference class (class 4). BMI, body mass index; CI, confidence interval; Glu, glucose; LDL-C, low-density lipoprotein cholesterol; HDL-C, high-density lipoprotein cholesterol; TC, total cholesterol; TG, triglycerides; Cr, creatinine; BUN, blood urea nitrogen.

Figure S5. Interaction between baseline pTau217, pTau217-lipid composite indices, and BP trajectories on dementia: Sensitivity analysis in nested case-control study

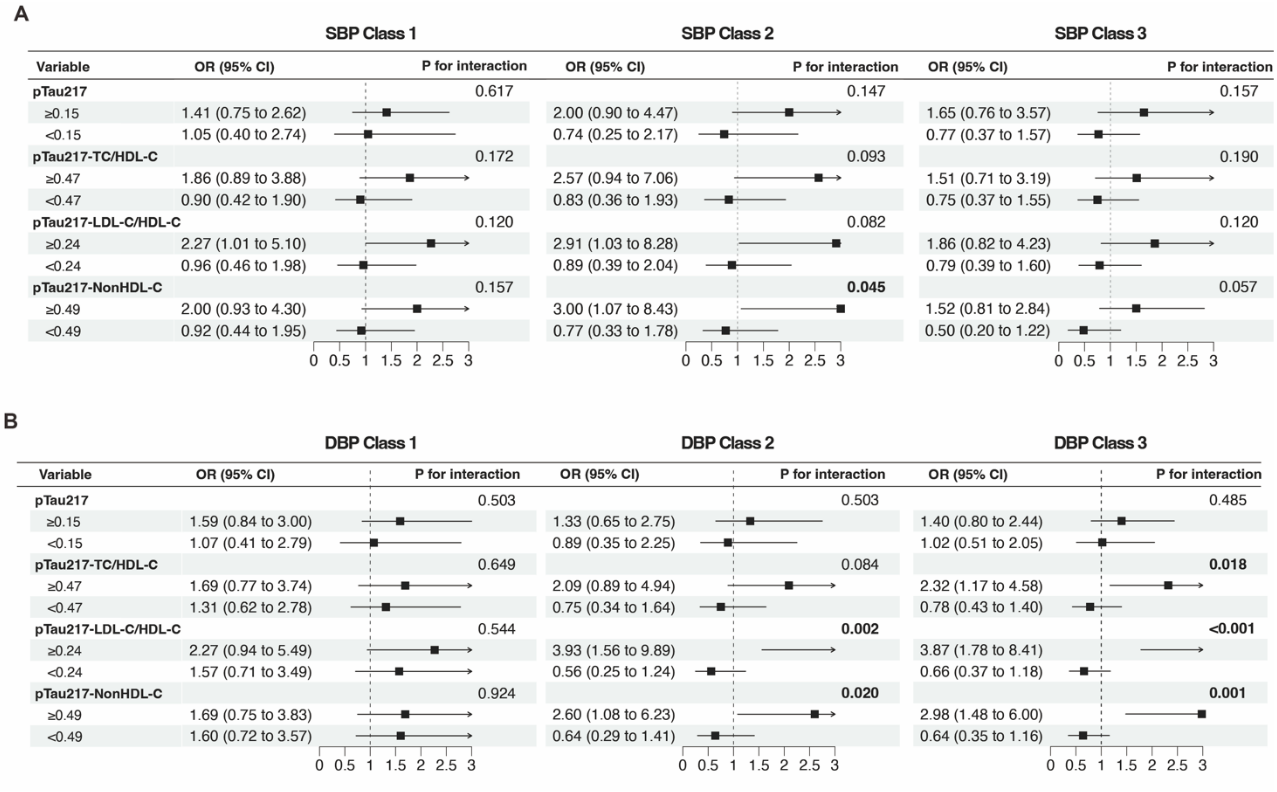

Odds ratios (OR) and 95% confidence intervals (CI) for the interactions between different SBP (A) and DBP (B) trajectory classes and baseline pTau217 and composite indices were estimated using generalized linear mixed models with random intercepts for village (cluster) and matched pair, accounting for clustering at the village and pair levels. Data are presented for BP trajectory classes 1-3, with class 4 as the reference. P values for interactions are shown for each model.
